# Cost-utility analysis of antihypertensive medications in Nigeria: a decision analysis

**DOI:** 10.1186/1478-7547-11-2

**Published:** 2013-01-23

**Authors:** Obinna Ikechukwu Ekwunife, Charles E Okafor, Charles C Ezenduka, Patrick O Udeogaranya

**Affiliations:** 1 Department of Clinical Pharmacy and Pharmacy Management, Faculty of Pharmaceutical Sciences, University of Nigeria Nsukka, 410001, Enugu, Nigeria; 2 Health Policy Research Group, University of Nigeria, Enugu Campus, P.O Box 1414, Enugu, Nigeria

## Abstract

**Background:**

Many drugs are available for control of hypertension and its sequels in Nigeria but some are not affordable for majority of the populace. This serious pharmacoeconomic question has to be answered by the nation’s health economists. The objective of this study was to evaluate the cost-effectiveness of drugs from 4 classes of antihypertensive medications commonly used in Nigeria in management of hypertension without compelling indication to use a particular antihypertensive drug.

**Methods:**

The study employed decision analytic modeling. Interventions were obtained from a meta-analysis. The Markov process model calculated clinical outcomes and costs during a life cycle of 30 years of 1000 hypertensive patients stratified by 3 cardiovascular risk groups, under the alternative intervention scenarios. Quality adjusted life year (QALY) was used to quantify clinical outcome. The average cost of treatment for the 1000 patient was tracked over the Markov cycle model of the alternative interventions and results were presented in 2010 US Dollars. Probabilistic cost-effectiveness analysis was performed using Monte Carlo simulation, and results presented as cost-effectiveness acceptability frontiers. Expected value of perfect information (EVPI) and expected value of parameter perfect information (EVPPI) analyses were also conducted for the hypothetical population.

**Results:**

Thiazide diuretic was the most cost-effective option across the 3 cardiovascular risk groups. Calcium channel blocker was the second best for Moderate risk and high risk with a willingness to pay of at least 2000$/QALY. The result was robust since it was insensitive to the parameters alteration.

**Conclusions:**

The result of this study showed that thiazide diuretic followed by calcium channel blocker could be a feasible strategy in order to ensure that patients in Nigeria with hypertension are better controlled.

## Introduction

Hypertension is a disturbance in hemodynamic function in which there is persistent abnormal elevation of systemic blood pressure, whether it is diastolic or systolic above the level of normal pressure of 140/90 mmHg [1]. It is regarded as a silent killer. Hypertension has a relationship with other cardiovascular diseases. Increasing blood pressure increases the risk of developing other cardiovascular diseases like stroke or coronary heart disease (CHD) [2].

There is growing evidence that prevalence of hypertension is on the increase in most sub-Saharan African countries including Nigeria [3]. A Meta analysis of prevalence rate of hypertension in Nigerian populations ranged from a minimum of 12.4% to a maximum of 34.8% with a combined prevalence of 22% [4]. With increasing prevalence and poorer control of hypertension, many people will be predisposed to cardiovascular events such as CHD and stroke. Such cardiovascular disease events will place a huge economic burden on the Nigerian economy since they are expensive to manage. Many drugs are now available for control of hypertension and its sequels but some are not affordable for majority of people in Nigeria. This serious pharmacoeconomic question has to be answered by the nation’s health economists.

Recent hypertension guidelines stress the usefulness of 4 classes of antihypertensive drugs i.e. thiazide diuretic, beta-blockers (BBs), angiotensin converting enzyme inhibitors (ACEIs), and calcium channel blockers (CCBs) which have been shown to be very effective compared to the others [5]. These drugs happened to be the predominant antihypertensive agents used in Nigeria. Therefore, the objective of this study was to evaluate the cost-effectiveness of drugs from these 4 classes of antihypertensive medications for use in management of hypertension in Nigerians without compelling indication to use a particular antihypertensive drug.

## Methods

### Effect sizes of hypertensive medication

The hypertensive medication selected for evaluation was based on a published systematic review whose aim was to evaluate the medication which should be first-line drug of choice for hypertension [5]. The study included randomized trials of at least one year duration, comparing one of 4 major classes of antihypertensive drugs (Table 1). More than 70% of the population of interest had BP > 140/90 mmHg at baseline [5]. The medications were classified as thiazide diuretic; BB; ACEI; and CCB. For the purpose of our study, hydrochlorothiazide, propranolol, lisinopril and nifedipine were the representative drugs for thiazide diuretic, BB, ACEI and CCB respectively as these drugs are commonly used in Nigeria [6].

**Table 1:** Summary of Interventions for improving control of hypertension

**Drug class**	**Drug**	**Dosage (daily)**
Thiazide diuretic	Hydrochlorothiazide	Tab. 25 mg
Beta blocker (BB)	Propranolol	Tab. 40 mg qid
Angiotensin converting enzyme inhibitor (ACEI)	Lisinopril	Tab. 10 mg
Calcium channel blocker (CCB)	Nifedipine	Tab. 10 mg

### Markov model

**Figure 1:**
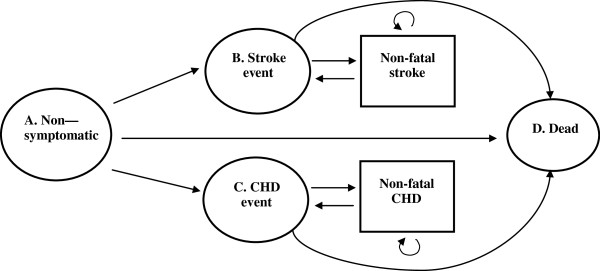
The life cycle model used to calculate the costs, effects and cost-effectiveness of the alternative interventions to manage hypertension.

The model life cycle used to calculate the cost, effect and cost-effectiveness of the alternative interventions to manage hypertension is shown in Figure 1. This cohort simulation (Markov process model) calculated clinical outcomes and costs during a life cycle of 30 years for 1000 people under the alternative intervention scenarios. The starting age of hypertensive patients in the model was 40 years. It was assumed that these hypertensive patients will start as asymptomatic and with each year that passes (Markov state), people may remain asymptomatic; they may experience any of the two major cardiovascular events which are stroke and coronary heart disease (CHD); they may remain in stroke or CHD (non-fatal); they may die from cardiovascular disease or may die from other causes not related to cardiovascular disease. The probabilities of these outcomes depended on the risk profile (as shown in Tables 2 and 3). Case fatality rates for stroke and CHD were 27% and 51% respectively [7].

**Table 2:** Index patients, with annual risks of CHD and stroke

**Age**	**Low risk**				**Medium risk**				**High risk**			
	**High BP**		**Controlled BP**		**High BP**		**Controlled BP**		**High BP**		**Controlled BP**	
	**CHD**	**Stroke**	**CHD**	**Stroke**	**CHD**	**Stroke**	**CHD**	**Stroke**	**CHD**	**Stroke**	**CHD**	**Stroke**
**40**	0.0010	0.0041	0.0010	0.0030	0.0041	0.0105	0.0030	0.0051	0.0072	0.0221	0.0041	0.0094
**45**	0.0020	0.0062	0.0010	0.0041	0.0051	0.0150	0.0041	0.0072	0.0094	0.0323	0.0062	0.0127
**50**	0.0030	0.0094	0.0020	0.0051	0.0072	0.0221	0.0051	0.0105	0.0138	0.0467	0.0083	0.0185
**55**	0.0041	0.0138	0.0030	0.0083	0.0105	0.0310	0.0072	0.0150	0.0185	0.0670	0.0116	0.0271
**60**	0.0051	0.0196	0.0041	0.0116	0.0150	0.0452	0.0105	0.0221	0.0271	0.0946	0.0161	0.0393
**65**	0.0072	0.0284	0.0062	0.0161	0.0209	0.0651	0.0150	0.0323	0.0378	0.1330	0.0233	0.0563
**70**	0.0105	0.0407	0.0083	0.0233	0.0297	0.0922	0.0209	0.0452	0.0530	0.1846	0.0323	0.0809

Total Cholesterol – 4.76 ± 1.06.

HDL Cholesterol – 0.90 ± 0.25.

Smoker – Low Risk = No; Medium Risk = No; High Risk = Yes.

**Table 3:** Nigerian Life Table – Yearly age specific mortality rate (29)

**Age**	**Index**	**Mortality rate**
**40-44**	40	0.01372
**45-49**	45	0.01529
**50-54**	50	0.01900
**55-59**	55	0.02569
**60-64**	60	0.03287
**65-69**	65	0.04781
**70-74**	70	0.07221
**75-79**	75	0.10773
**80-84**	80	0.15920
**85-89**	85	0.22886
**90 and above**	90	0.32027

Quality Adjusted Life Years (QALYs) was used to quantify clinical outcome. The health related quality of life weights for the different states (non-symptomatic, stroke, and CHD) were obtained from the data of a health utility state assessment carried out in Nigeria [8]. To obtain the QALYs which represent the clinical outcome, the weights were multiplied by the number of life years spent in the different states, and then averaged over a life time of 30 years with a utility discount of 3%. This was done for the different interventions. The average cost of treatment for the 1000 patients was tracked over the Markov cycle model for all the interventions including null scenario. The average QALYs and costs were calculated by summing up the QALYs and costs respectively over a period of 30 years and then dividing by the population (1000 patients). Financial values were presented in 2010 US dollars. Discount of 3% at baseline were use for both cost and effects.

A Null scenario was included in this model. The null scenario refers to the case where the patients at asymptomatic stage (1000 patients) are not receiving any of the antihypertensive medications under evaluation. The null scenario however, included the cost of management and the cost of hospitalization for the hypertension-related complications (stroke and CHD). The reason for including the null scenario in this model is to calculate the extra cost that will be incurred if the patients began to treat their hypertension at asymptomatic stage with any of the medications under study and also to evaluate if the patients in their asymptomatic stage who began treatment with any of the antihypertensive drugs will have a higher or lower quality of life years (QALYs) when compared to the case where the patients at their asymptomatic stage received no treatment (null scenario) until they had stroke or CHD. The extra cost (incremental cost) and the difference in QALY was used to evaluate which of the antihypertensive agents or null scenario is the most cost-effective.

### Calculating risk of cardiovascular event

A web-based recalibrated Framingham risk score designed for estimating 10-year risk of stroke and CHD in seven British black and minority group was used to estimate the risk of stroke and CHD of the different age group [9]. Since the recalibration of the risk scores was done with Africans in Britain, its use in this model will give a better risk prediction as there was no published study that predicted cardiovascular events over time in Africans that could be used for this model. The World Health Organization/International Society of Hypertension cardiovascular risk stratification (low risk, medium risk and high risk) was used to stratify the patients [10]. Index patients used for low risk patients were such that their highest 10 years risk of stroke or CHD were less than 15% at the age of 50. Index patients used to depict medium risk had 10 years stroke or CHD risk of 15% to 20% at the age of 50 while high risk patients had 10 years stroke or CHD risk of greater than 20% at the age of 50. Average values of total cholesterol and high density lipoprotein (HDL) in Nigerians were used for stroke and CHD risk calculation. The values were obtained from a prospective population survey conducted in Port Harcourt, a city located in southern part of Nigeria [11]. The 10 years risk of stroke or CHD was converted to one year risk (annual transition probabilities) [12].

### Determination of cost

Cost was estimated from the providers’ perspective (third party payer). Providers’ perspective requires mainly the direct cost to be measured [13]. In this study, the direct cost includes cost of medications; cost of physician visit; cost of in-patient stay; cost of physiotherapy; and cost of laboratory tests. Cost of medications were gotten from the Nigerian National Health Insurance Scheme (NHIS) drug price list, published in 2005 [14], except for Mannitol (20% IVF) whose cost was gotten from the 2010 International Drug Price Indicator Guide of the WHO [12]. Cost of in-patient stay, physician visit (1st and review), computed tomography scan, echocardiography, electrocardiography and urinalysis were also obtained from the NHIS drug price list [14]. Cost gotten from the 2005 NHIS drug price list were adjusted to reflect the future (2010) value using a real interest rate of 3% (range of 0% - 5%). For cost obtained from the International Drug Price Index (Mannitol), the median price was used and multiplied by the Power Purchasing Parity Exchange Rate (PPP) to obtain the value in Naira which was then divided by the yearly exchange rate to obtain the US dollar equivalent [15,16]. The cost of magnetic resonance imaging (MRI), full blood count (FBC) and serum electrolyte and urea analysis (SEU) were gotten from the price list of University of Nigeria Teaching Hospital (UNTH), Ituku-Ozalla.

For patients at the asymptomatic hypertension stage, their direct costs include cost of any of the antihypertensive medication under study and cost of physician visit. It was assumed that the medications are taken daily for 365 days in a year while physician visit was made to be monthly (i.e. 12 times per annum). For Stroke patients hospitalized, the direct costs used in the model were cost of medical therapy (mannitol and furosemide), in-patient stay, laboratory tests and physiotherapy. For CHD patients hospitalized, the direct costs used in the model were cost of medical therapy (nifedipine and nitroglycerin), in-patient stay and laboratory tests. The costs were calculated as annual costs under the following headings: cost for use of thiazide; cost for use of lisinopril; cost for use of nifedipine; cost for use of propranolol; cost to hospitalize stroke patients; cost to manage stroke patients; cost to hospitalize CHD patients; and cost to manage CHD patients. A triangular distribution was used to calculate the annual costs under the headings listed above.

The cost of providing health care to hypertensive patients (asymptomatic) and those with hypertension-related complications in the null was estimated first before determining the incremental cost that will be incurred if the patients from their asymptomatic stage to hypertension-related complications (stroke and CHD) had consume any of the four antihypertensive drugs (propranolol, HCTZ, Nifedipine and Lisinopril) under evaluation. The summary of the interventions cost are shown in Table 4.

**Table 4:** Data Inputs and distribution in Markov model

**Variable**	**Mean/Mode**	**Distribution**	**Source**
*Probabilities*			
Probabilities of non-symptomatic to stroke or CHD	See Table 2	Gamma	6
Probability from stroke to Death	0.27	Beta (α 0.27, β 0.73)	29
Probability from CHD to Death	0.51	Beta (α 0.49, β 0.51)	29
*Relative Risk*			
Relative Risk of Thiazide to Stroke	0.63	Normal (α 0.63, se 0.056)	4
Relative Risk of Thiazide to CHD	0.84	Normal (α 0.84, se 0.060)	4
Relative Risk of Thiazide to Death	0.89	Normal (α 0.89, se 0.037)	4
Relative Risk of Propranolol to Stroke	0.83	Normal (α 0.83, se 0.076)	4
Relative Risk of Propranolol to CHD	0.90	Normal (α 0.90, se 0.071)	4
Relative Risk of Propranolol to Death	0.96	Normal (α 0.96, se 0.056)	4
Relative Risk of Lisinopril to Stroke	0.65	Normal (α 0.65, se 0.116)	4
Relative Risk of Lisinopril to CHD	0.81	Normal (α 0.81, se 0.075)	4
Relative Risk of Lisinopril to Death	0.83	Normal (α 0.83, se 0.071)	4
Relative Risk of Nifedipine to Stroke	0.58	Normal (α 0.58, se 0.183)	4
Relative Risk of Nifedipine to CHD	0.77	Normal (α 0.77, se 0.174)	4
Relative Risk of Nifedipine to Death	0.86	Normal (α 0.86, se 0.120)	4
*Resources (Annual cost/patient in US Dollars)*			
Thiazide	89.69	Triangular (min 71.75, max 107.63)	11
Propranolol	103.36	Triangular (min 82.69, max 124.03)	11
Lisinopril	404.08	Triangular (min 323.26, max 484.89)	11
Nifedipine	294.72	Triangular (min 235.78, max 353.67)	11
Hospitalization cost for Stroke	873.62	Triangular (min 698.89, max 1048.34)	11,12,UNTH
Hospitalization cost for CHD	311.73	Triangular (min 249.39, max 374.08)	11, UNTH
Management of Stroke and CHD Patients	417.74	Triangular (min 334.20, max 501.29)	11
*Health Utilities*			
Hypertensive patients	0.57	Beta (α 206.1, β 155.1)	Unpublished
Stroke Patients	0.04	Beta (α 1.0, β 23.3)	Unpublished
CHD	0.13	Beta (α 4.9, β 32.9)	Unpublished
*Discount Rate*			
Cost and Utility	3%	Triangular (min 0%, max 5%)	30

### Handling uncertainty

Distributions appropriate for each variable were employed in order to capture the varying degree of inherent uncertainty in the variables used in the analysis (Table 5). Probabilistic sensitivity analysis was used to assess simultaneous uncertainty in many variables. This approach is well suited to express overall parameter uncertainty [9]. A total of 1000 iterations of Monte Carlo simulations were conducted and for each iteration, a value was drawn randomly from each distribution and net health benefits calculated [9].

**Table 5:** Expected Value of Perfect Information (1000 Patients, 30 years)

Intervention*	**Willingness to pay ($/QALY)**	**Population EVPI ($)**
*Low Risk*		
Null Scenario	**-**	-
Thiazide diuretic	2,700	2,066,053
ACEI	15,000	18,246,980
*Medium Risk*		
Null Scenario	-	-
Thiazide diuretic	1,300	1,235,304
CCB	15,000	38,307,837
*High Risk*		
Null Scenario	-	-
Thiazide	1,400	894,629
CCB	12,500	22,299,390

*Intervention are presented in decreasing order of cost-effectiveness.

ACEI – Angiotensin converting enzyme inhibitor; CCB – Calcium channel blocker.

### Net monetary benefit calculation

The application of net monetary benefit (NMB) approach has its importance in overcoming the problems associated with incremental cost-effectiveness ratios (ICERs) [17]. This was obtained by applying the formula below:

(1) $$\text{NMB}=\mathrm{\Delta QAL}Y_{i}-\left(\mathrm{\Delta Cos}t_{i}/\lambda \right)$$

Where NMB = Net monetary benefit

QALY = Quality adjusted life years

λ = Threshold ratio

In the net monetary benefit approach, a strategy or intervention is considered cost-effective if it produces a positive net benefit [12]. On the other hand, a negative net benefit means that the intervention is considered not to be cost-effective and as such, will not be considered. For each iteration, the intervention with the highest NMB is designated the value ‘1’ while other intervention for that iteration will the value ‘0’. After 1000 iterations, the values (1 or 0) for each intervention are summed and averaged to obtain their probabilities of cost-effectiveness.

The cost-effectiveness threshold (CET) used in this evaluation was $15,000 which is the maximum amount the providers’ will be willing to pay, to achieve the best therapeutic outcome for their clients. The CET used was just an arbitrary figure within range that will aid to obtain the final result.

### Cost-effectiveness acceptability frontier

Cost-effectiveness acceptability frontiers were used to present the result of cost-effectiveness for the 3 different cardiovascular risk scenarios. The cost-effectiveness acceptability frontiers illustrate the probability of any intervention being optimal compared to all other competing alternatives. Cost-effectiveness frontier also illustrates the crossover when one intervention is substituted by the other as the one with the highest probability of being optimal and therefore provides useful information for policy makers [18]. The major difference between the frontier and curve is that the frontier takes into consideration the null scenarios in addition to the alternative interventions while the acceptability curve considers all the interventions except the null scenario. A total of 58 iterations of simulations were conducted for different willingness-to-pay threshold ratio. For each iteration, the probability that the cost-effectiveness of any intervention being optimal compared to the null scenario was calculated for all the alternative interventions from the NMB [12].

### Assessment of population values of perfect information (EVPI)

Population Expected Value of Perfect Information (EVPI) was carried out for the three cardiovascular risk scenarios to determine the opportunity cost surrounding the conclusion of the analysis [12]. The population EVPI was conducted for 1000 patients over the life cycle of 30 years period. Simulation for 58 iterations were conducted for different willingness-to-pay threshold ratio and for each iteration, a value of perfect information for each willingness-to-pay threshold was calculated with the use of the effective population [12].

Across the four anti-HTs under evaluation including null scenario, the highest NMB for each iteration was selected. The highest NMBs for all the iterations were averaged. The difference between this average value obtained and the highest averaged NMB across the different interventions was noted. In calculating the Discount Population for the 1000 patients over a period of 30 years, a discount rate of 3% (range of 0% - 5%) was used. The sum of the discount population over 30 years is the Effective Population. The Population EVPI was obtained by multiplying the Effective Population with the difference in NMB noted.

### Assessment of parameter value of perfect information (EVPPI)

The expected value of perfect information for parameters (EVPPI) was obtained by multiplying the effective population with the difference between the net-benefit with perfect information and the expected value with current information about the parameter(s) [12]. The EVPPI was also conducted for 1000 patients. This assessment was started by checking the threshold ratio and set to the maximum in order to give a high EVPI. The parameters assessed were relative risk (RR), utility and cost. A total of 100 iterations of Monte Carlo simulation were conducted and for each parameter, the net-benefit was obtained 10 times by repeating the process so as to calculate the mean net-value. The perfect information pay-off, given certainty over each parameter was determined in order to obtain the EVPPI.

### Data analyses

All analyses (i.e. Markov chain analysis, Monte Carlo simulation, and assessment of value of perfect information) were carried out using Microsoft Excel (Microsoft Corporation, 2007).

## Results

For the three cardiovascular risk groups, the yearly cost in the null scenario was the least. The null scenario also had the least QALYs in the three groups. In all the cardiovascular risk states (CVRSs), ACEI has the highest annual cost if used followed by calcium-channel blocker. ACEI has a low QALY in the medium and high risk state but the highest QALY in the low CVRS. Thiazide diuretic has a very low annual cost in the three states, the highest QALYs in the medium and high risk state but not in the low CVRS. In the low CVRS, at any threshold ratio ($0 - $15,000), ACEI has the highest NMB followed by thiazide but in the medium and high CVRSs, thiazide has the highest NMB followed by calcium-channel blocker at any threshold ratio. For instance, in the medium risk, at threshold ratio of $15,000, the NMB of the various interventions are as follows: null scenario ($89799); thiazide ($101927); beta-blocker ($94094); ACEI ($98619); Calcium-channel blocker ($101780).

The null scenario in the three cardiovascular risk groups has a 0 (zero) probability of being cost effective. In the low CVRS, ACEI has the highest probability of being cost effective (about 0.526) followed by thiazide diuretic (about 0.278). In the medium and high CVRSs, calcium-channel blocker has the highest probability of being cost effective (about 0.47 and 0.53) followed by thiazide diuretic (about 0.45 and 0.41) respectively.

**Figure 2:**
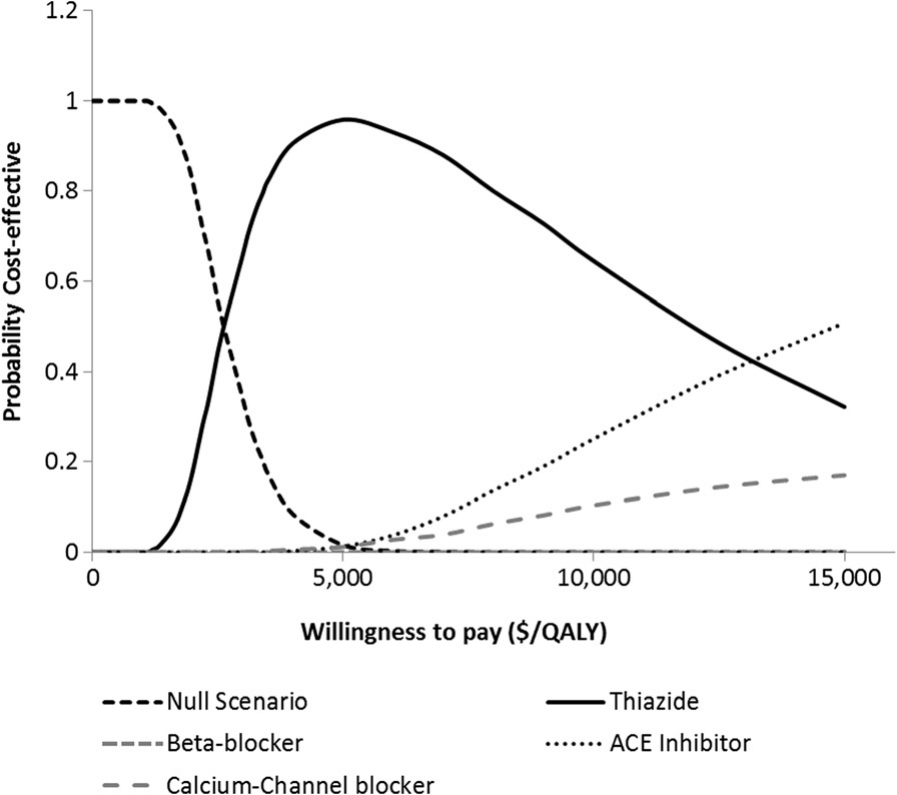
Cost-effectiveness acceptability frontier for the alternative interventions in the low cardiovascular risk scenario.

**Figure 3:**
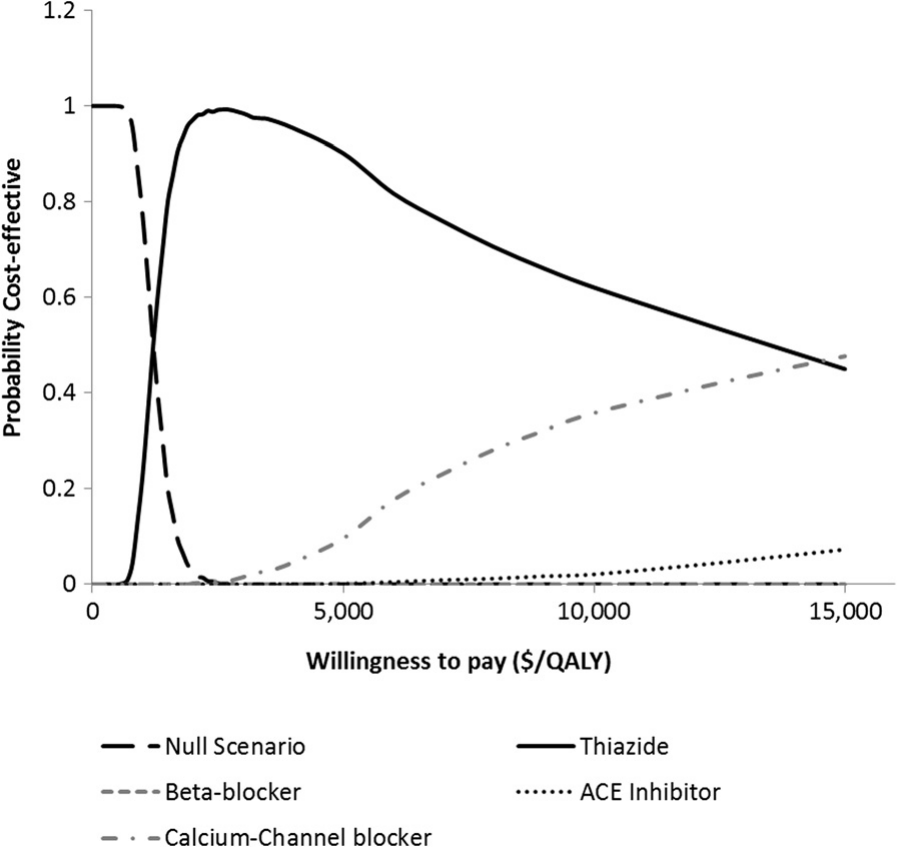
Cost-effectiveness acceptability frontier for the alternative interventions in the moderate cardiovascular risk scenario.

**Figure 4:**
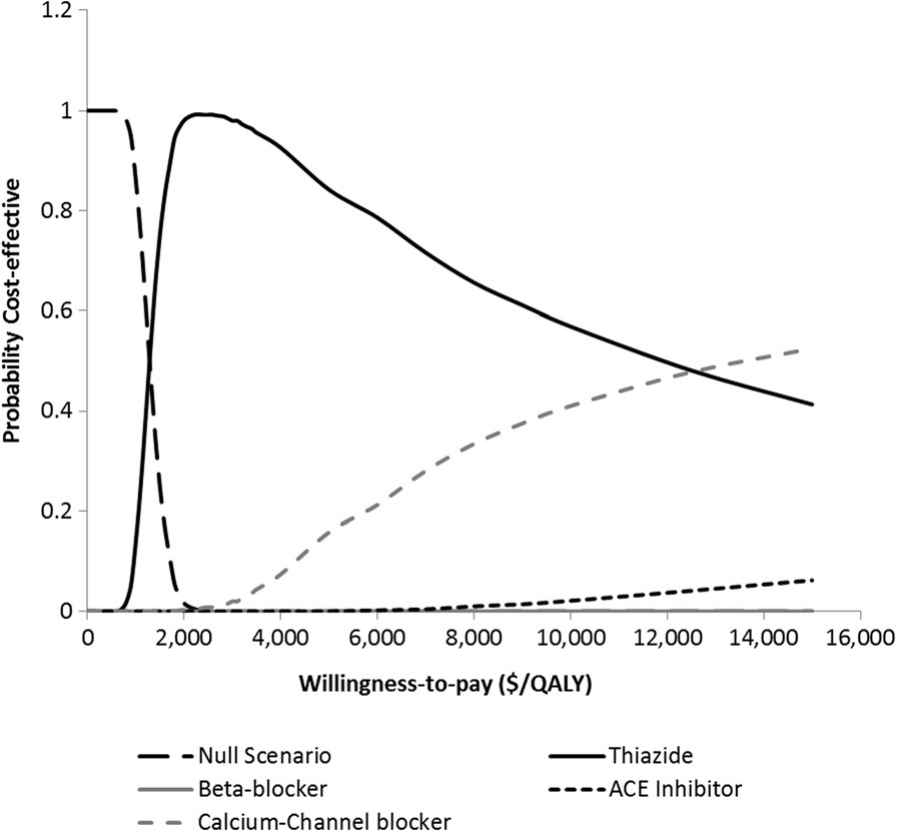
Cost-effectiveness acceptability frontier for the alternative interventions in the high cardiovascular risk scenario.

The application of cost-effectiveness acceptability frontier is to help determine which antihypertensive agent has the highest probability of being cost-effective when compared with other alternative drugs. For low cardiovascular risk state (Figure 2), at no willingness-to-pay, null scenario was the most cost-effective (if the provider is not willing to invest any money to achieve a higher health outcome). When the provider is ready to pay any amount above $2,600 but not more than $15,000 for additional QALY, thiazide diuretic emerged as the best intervention. If the provider is willing to pay greater than $15,000 for additional QALY, ACEI emerged as the best intervention. In Figure 3, cost-effectiveness frontier for patients in medium cardiovascular state is shown. At no willingness-to-pay, null scenario was the most cost-effective. When the provider is ready to pay any amount above $1,300 but not more than $15,000 for additional QALY, thiazide emerged as the best intervention. If the provider is willing to pay greater than $15,000 for additional QALY, calcium-channel blocker emerged as the best intervention. Similar result was also obtained for patient in high cardiovascular risk state (Figure 4). At no willingness-to-pay, null scenario was the most cost-effective. When the provider is ready to pay any amount above $1,300 but not more than $15,000 for additional QALY, thiazide emerged as the best intervention. If the provider is willing to pay greater than $12,500 for additional QALY, calcium-channel blocker emerged as the best intervention.

Population expected value of perfect information analysis (EVPI) in Table 5 showed that the opportunity cost surrounding the decision to implement any of the intervention that emerged cost-effective at a given willingness-to-pay threshold ranged from approximately $26,000,000 to $73,000,000. This range also implies the maximum that the providers will be willing to pay in order to obtain perfect information so as to obtain a perfect result. In Table 5, the interventions shown were the interventions which at a particular willingness-to-pay, becomes the most cost effective. For any of the risk states, interventions which are not cost-effective at any point are not included in the table. The willingness-to-pay used in Table 5 is the point from which an intervention emerges as the most cost-effective for a particular risk state.

Analysis for the expected value of perfect information for parameters (EVPPI) in Table 6 showed that the population EVPPI for the various parameters was far below the population EVPI, indicating that the providers can easily pay-off such amount to obtain perfect information of the parameters. Parameters such as cost of managing stroke patients and yearly cost of using thiazide diuretic has negligible EVPPI for low risk and high risk state.

**Table 6:** Expected Value of Perfect Information for Parameters

**Parameter**	**Population EVPPI ($)**	**Population EVPI ($)**
**Low Risk**		27,076,912
*Relative Risk of Thiazide*		
Stroke	-	
CHD	-	
Death	-	
*Utility*		
Non-symptomatic	5,576	
Stroke	2,938	
CHD	5,006	
*Cost*		
Stroke Hospitalization	16,518	
CHD Hospitalization	14,984	
Stroke Manage	-	
Yearly Cost Thiazide	178	
**Moderate Risk**		73,353,965
*Relative Risk of Thiazide*		
Stroke	3,093,981	
CHD	2,648,842	
Death	7,221,002	
*Utility*		
Non-symptomatic	2,092,005	
Stroke	3,003,524	
CHD	2,303,848	
*Cost*		
Stroke Hospitalization	1,224,642	
CHD Hospitalization	1,781,143	
Stroke Manage	2,392,084	
Yearly Cost Thiazide	4,126,641	
**High Risk**		26,667,500
*Relative Risk of Thiazide*		
Stroke	9,901,387	
CHD	5,441,116	
Death	646,992	
*Utility*		
Non-symptomatic	1,137,622	
Stroke	2,005,656	
CHD	1,336,286	
*Cost*		
Stroke Hospitalization	1,378,708	
CHD Hospitalization	2,908,565	
Stroke Manage	660,668	
Yearly Cost Thiazide	437,657	

## Discussion

From the result, thiazide diuretic among the four alternatives was the most cost-effective. This finding was consistent for the 3 cardiovascular risk scenarios. For low risk patients, lisinopril was the second most cost-effective option to implement but additional fund needs to be committed in order to achieve better health outcomes over thiazide diuretic. CCB was the second most cost-effective option for medium and high risk patients if additional fund is committed in order to achieve better health outcomes over thiazide diuretic. In the graphs, the probability that thiazide diuretic is cost-effective decreases as the CE threshold increases. This is so because beyond a willingness-to-pay of about $2,600/QALY, committing more funds does not yield a corresponding increase in QALY for thiazide. So, additional fund beyond $15,000/QALY is a waste of resource since there is no increase in clinical outcome. Beyond that limit, CCB should be considered.

From the report of a recent Meta analysis aimed to quantify the benefits and harm of the major first-line anti-hypertensive drug classes, thiazide diuretic was the best choice for hypertension [5]. Most of the evidence demonstrated that first-line low dose thiazide diuretic reduces mortality and morbidity (stroke, heart attack and heart failure). No other drug improved health outcomes better than low-dose thiazide [5]. This analysis shows that with cost consideration, low dose thiazide diuretic is very cost-effective. Thus, in low resource settings such as in Nigeria and other developing countries, thiazide diuretic should be considered the drug of first choice especially in patients that respond well to it and those that do not have other co-morbidities that will necessitate the use of a particular antihypertensive agent.

That an intervention is most cost-effective depends on what the providers are willing to pay per outcome. As a guide, WHO considers interventions to be cost-effective if they have incremental cost-effectiveness ratios (ICERs) that are less than three times the gross national income (GNI) per capita [19]. It is pertinent to state that at 50% probability of cost-effectiveness, the willingness-to-pay is less than three times the GNI per capita. The national income per capita for Nigeria in 2009 was USD1, 190 or USD3, 370 when multiplied by three. Based on the above premise, thiazide diuretic could be judged a cost-effective option.

From the University of Toronto, in a published cost-effectiveness analysis of routine echocardiography in patients starting antihypertensive drug therapy, the result of the model showed that ACE-inhibitors cannot be recommended as antihypertensive first-line therapy in the patients under study [20]. This was because ACE-inhibitors were very expensive and the gain in unadjusted and quality-adjusted life-years was small and clinically irrelevant [20]. From the author’s result, prescribing conventional antihypertensive therapies (diuretics and beta-blockers) to everybody can be recommended as strategies of choice [20]. This is in line with the result of this study because the result showed that ACE-inhibitors are very expensive with low QALYs and their ICERs are far more than three times the GNI of Nigeria.

However, the result of this study does not completely support the guideline by the Sixth Report of the Joint National committee on prevention, detection, evaluation and treatment of high blood pressure (JNC- 6) which recommends the use of diuretics and beta-blockers as first-line antihypertensive drugs in the absence of compelling reasons to use other antihypertensive drugs [21]. The result of this study does not also completely support the result in the cost-effectiveness study from the University of Toronto which recommended the prescribing of diuretics and beta-blockers to everybody [20]. This is evidently because, in the result of this study, beta-blocker was never a contender for cost-effectiveness. For Nigerians, in place of beta-blocker, calcium-channel-blocker should be considered a second-line therapy after diuretics.

In the context of research, with respect to this study, EVPI represent the maximum that providers can willingly pay for additional research, to inform the decision they make [22]. The EVPI analysis showed that the opportunity cost surrounding the choice of thiazide diuretic for all the cardiovascular risk state is lower compared to the choice to use ACEI or CCB. A reason for this is because the willingness-to-pay from which thiazide diuretic becomes most cost-effective is relatively small. This supports the fact that thiazide could be judged a cost-effective option compared to the other antihypertensive drugs. On the other hand, analysis for the expected value of perfect information for parameters (EVPPI) showed that there may not be need of further experimental research to get perfect parameter estimates since the population EVPPI for the various parameters was far below the population EVPI. Change in the parameters caused an infinitesimal changed in the result showing that the result is insensitive to the parameters which invariably means that the result is robust.

This analysis has some limitations which have to be considered when interpreting the results. In our Markov model to show a typical timeline of events consequent on hypertension, we made use of an algorithm designed from data obtained from Africans in Britain, which may not hold true for indigenous Africans and Nigerians in particular. The reason for use of those data in the model was because there was no published study that predicted cardiovascular events over time in Africans that could be used for this model.

In conclusion, the result of this study shows that thiazide diuretic followed by calcium-channel blocker especially for medium and high risk patients is a cost-effective option in the management of patients with high blood pressure in Nigeria.

## Competing interests

Funding for this research, a project in partial fulfillment of the requirement for the award of a Bachelor of Pharmacy (B.Pharm) was provided partly by parents and friends of Okafor, C.E. The authors declare no conflict of interest.

## Authors’ contributions

OIE, CEO and CCE designed the study. OIE designed the model while CEO and PO helped in gathering data. CEO wrote the first draft of the manuscript. All authors read and approved the final manuscript.
